# Preprocedural Anxiety in Kidney Biopsy: A Prospective Study of Prevalence, Risk Factors, and Physiological Correlates

**DOI:** 10.3390/jcm15020544

**Published:** 2026-01-09

**Authors:** Kittiphan Chienwichai, Sirin Jiwakanon, Kamonrat Chaiviriyawong, Jananya Wattanakul, Thanapong Sungworawongpana, Sorawat Sangkaew, Arunchai Chang, Pannawat Mongkolrattanakul, Songklod Pakdeejit

**Affiliations:** 1 Division of Nephrology, Department of Internal Medicine, Hatyai Hospital, Songkhla 90110, Thailand; 2 Department of Internal Medicine, Hatyai Hospital, Songkhla 90110, Thailand; 3 Department of Social Medicine, Hatyai Hospital, Songkhla 90110, Thailand; 4 Division of Gastroenterology, Department of Internal Medicine, Hatyai Hospital, Songkhla 90110, Thailand; 5 Division of Nephrology, Department of Internal Medicine, Phanatnikhom Hospital, Chonburi 20140, Thailand; 6 Division of Intervention Radiology, Department of Radiology, Hatyai Hospital, Songkhla 90110, Thailand

**Keywords:** anxiety, blood pressure, kidney biopsy, prevalence

## Abstract

**Background/Objectives:** Despite the widespread recognition of preprocedural anxiety in awake invasive procedures, there is a paucity of data examining its prevalence and clinical impact in patients undergoing percutaneous kidney biopsy. This study aimed to determine the prevalence of preprocedural anxiety, assess its association with peri-procedural hemodynamic parameters, and identify factors associated with elevated anxiety. **Methods**: In this prospective observational study, 151 adults scheduled for percutaneous kidney biopsy between June 2023 and January 2025 were enrolled. Anxiety was assessed 24 h before the procedure using the Thai State–Trait Anxiety Inventory Y1 (STAI-Y1). Blood pressure and pulse rate were measured at baseline and 30 min before biopsy. Mixed-effects models evaluated associations between anxiety and hemodynamic changes, and logistic regression identified predictors of anxiety. **Results**: Clinically significant anxiety (STAI-Y1 ≥ 40) was present in 55% of patients, with 43.4% reporting very high anxiety. Anxiety status was not independently associated with changes in systolic or diastolic blood pressure or pulse rate. However, diastolic blood pressure increased significantly from baseline to preprocedural across all patients (mean increase 5.45 mmHg; *p* = 0.008), irrespective of anxiety. Higher serum creatinine (OR 1.29; *p* = 0.012) and a history of previous kidney biopsy (OR 4.28; *p* = 0.004) were independently associated with anxiety. **Conclusions**: Preprocedural anxiety is highly prevalent among patients undergoing kidney biopsy but does not independently influence peri-procedural hemodynamic parameters. Targeted screening and supportive interventions may benefit patients at increased risk of anxiety.

## 1. Introduction

Despite advances in non-invasive biochemical and imaging investigations in clinical nephrology, percutaneous kidney biopsy remains the gold standard for diagnosing various kidney diseases [1,2]. Although generally safe, kidney biopsies are invasive procedures commonly performed under local anesthesia while the patient is awake. The nature of this procedure can provoke significant stress and anxiety.

Anxiety, defined as a subjective state of emotional uneasiness or fear accompanied by somatic symptoms and autonomic changes that can impair normal functioning [3], is common but often under-recognized in clinical nephrology. Although anxiety related to other nephrology procedures, such as arteriovenous fistula (AVF) creation [4] and AVF cannulation [5], has been documented, evidence specific to kidney biopsy-induced anxiety remains limited.

In the context of a kidney biopsy, anxiety may affect not only psychological well-being but also physiological responses, such as elevated blood pressure and heart rate [6,7,8], which are known to increase the risk of post-biopsy complications [9]. Although clinical guidelines recommend ultrasound-guided biopsies to reduce procedural complications [10], complications such as pain, hematoma, hematuria, and, rarely, the need for transfusion or death still occur, with an estimated mortality rate of 0.06% [11]. Given the increasing frequency of kidney biopsies worldwide [12,13], with annual incidence rates ranging from 10 to 200 per million people [14], recognizing and managing preprocedural anxiety has become increasingly important.

Despite evidence that preprocedural anxiety varies according to factors such as gender [6], preoperative information [6], type of procedure [15], and socioeconomic status [16,17], these factors have not been systematically examined in patients undergoing kidney biopsy. Characterizing anxiety predictors in this specific population would enable clinicians to identify high-risk patients and implement tailored anxiolytic strategies during preoperative preparation.

This study aimed to investigate the prevalence of preprocedural anxiety in patients undergoing kidney biopsy at a tertiary care center in Thailand. Additionally, it explores the association between anxiety and demographic factors and the impact of anxiety on hemodynamic parameters. We hypothesize that anxiety levels before a kidney biopsy are high and comparable to levels observed in patients awaiting surgery, likely influenced by concerns about kidney disease and bleeding risks associated with the procedure.

## 2. Materials and Methods

Adult patients (aged 18 years and older) admitted for percutaneous kidney biopsy were included in this prospective observational study, conducted from June 2023 to January 2025 at the Department of Nephrology, Hatyai Hospital, Thailand. The exclusion criteria included the inability to complete a self-assessment questionnaire, a history of kidney transplant, pregnancy, and the use of anxiolytic medications within 72 h before the assessment. Additionally, patients with pre-existing psychiatric conditions, such as anxiety disorders or depression, were excluded from the study. Psychiatric conditions were identified based on medical records and patient self-reports during the admission process.

This study is reported in accordance with the Strengthening the Reporting of Observational Studies in Epidemiology (STROBE) statement [18], and the completed checklist is provided as an Additional file. The study was conducted in accordance with the ethical principles outlined in the Declaration of Helsinki. All patient data were analyzed anonymously, and written informed consent was obtained from all patients or their legal guardians, as applicable.

### 2.1. Kidney Biopsy Protocol

After the nephrologist assesses the clinical indications and contraindications for a kidney biopsy, the nurse coordinator schedules the procedure. A trained nurse then educates patients about the procedure, including its risks, benefits, and necessary care instructions. Patients are admitted one day before the biopsy, instructed to fast for 8 h overnight, and monitored for at least 24 h afterward.

An experienced interventional radiologist performs the biopsy using a 16-gauge automated spring-loaded device under real-time ultrasound guidance. At our center, premedication with desmopressin or anxiolytics is not part of routine practice; therefore, no patients received these medications before or after the procedure, regardless of renal function. Patients were positioned prone with a pillow under the abdomen to optimize kidney exposure. The prone position is the standard approach at our institution for all native kidney biopsies. The lower pole of the kidney was targeted to minimize the risk of vascular injury. After sterile preparation and draping, local anesthesia (2% lidocaine) was infiltrated from the skin to the renal capsule under real-time ultrasound guidance. The needle was advanced to the renal cortex, and two to three cores were obtained. Direct pressure was applied to the biopsy site for approximately 10 min after the procedure. Patients did not receive anxiolytics or sedation before or after the procedure. The patient’s blood pressure, cardiac rhythm, and oxygen saturation are monitored during the procedure. After the biopsy, patients remain in bed on their backs for a 24 h observation period. During this time, clinical signs (e.g., gross hematuria, flank pain, or hypotension) and ultrasound evaluations were performed as needed to identify potential bleeding complications. The nurse who measured blood pressure and recorded clinical data was blinded to the results of the anxiety assessment.

### 2.2. Hemodynamic Assessment

To evaluate the effect of anxiety on hemodynamic parameters, blood pressure and pulse rate were recorded during the patients’ outpatient visit before the kidney biopsy. These measurements were repeated 30 min before the procedure using an automated office blood pressure device, with the average of three readings used for analysis.

Continuous blood pressure monitoring and heart rate variability analysis were not performed due to resource constraints, including the lack of specialized equipment and additional personnel within our standard preprocedural workflow.

### 2.3. Anxiety Assessment

Anxiety was assessed 24 h before kidney biopsy using the State-Trait Anxiety Inventory Y1 (STAI-Y1) [19], self-administered by patients upon hospital admission, with completed forms sealed in envelopes and not reviewed by clinical staff until after data collection was complete. This time point was selected due to clinical workflow constraints, as patients are routinely admitted one day before the procedure for preprocedural preparation. The research nurse responsible for hemodynamic measurements remained blinded to participants’ anxiety scores throughout the study. We used the Thai version, which has been previously translated and validated [20], with demonstrated good internal consistency (Cronbach’s α = 0.88). The STAI-Y1 is the definitive instrument for measuring anxiety in adults [21] and has been used in several studies [22,23]. Furthermore, the inventory’s simplicity makes it ideal for evaluating individuals with lower educational backgrounds [21]. Subjects were asked to rate the intensity of their anxiety on 20 items using a self-report questionnaire. Each item was scored on a 4-point scale: “not at all,” “somewhat,” “moderately so,” or “very much so.” The total score ranged from 20 to 80. STAI-Y1 values of ≥40 defined the presence of clinically significant anxiety [24].

### 2.4. Statistical Analysis

Patients were categorized into two groups based on STAI-Y1 scores: the Anxiety group (STAI-Y1 ≥ 40) and the Non-Anxiety group (STAI-Y1 < 40). Categorical variables were presented as numbers and percentages, and differences between groups were assessed using the chi-square test or Fisher’s exact test, as appropriate. Continuous variables were summarized as means and standard deviations or as medians and interquartile ranges, depending on the normality of the distribution. Differences between groups were evaluated using Student’s *t*-test for normally distributed data or the Wilcoxon rank-sum test for non-normally distributed data.

To test the hypothesis that anxiety affects hemodynamic parameters—as measured by changes in blood pressure and pulse rate from the outpatient visit (baseline) to 30 min before the kidney biopsy (pre-biopsy)—we used a mixed-effects model for repeated measures (MMRM). Fixed effects in the model included anxiety group (Anxiety vs. Non-Anxiety), time (baseline vs. pre-biopsy), and covariates such as sex, age, BMI, serum creatinine, and use of antihypertensive medications. For the pulse rate analysis, beta-blocker use was included as an additional adjustment variable. An interaction term between anxiety group and time was incorporated to evaluate whether changes in blood pressure over time differed by anxiety status. A random intercept for each patient was included to account for within-subject correlation across repeated measures, with an unstructured variance-covariance matrix specified to model the correlation structure. This approach enabled us to assess whether anxiety levels significantly influenced the change in blood pressure from baseline to pre-biopsy while controlling for potential confounders.

To investigate factors associated with anxiety, we employed a logistic regression model. Variables with a *p*-value < 0.1 in univariate analyses (including sex, age, and other relevant factors) were subsequently entered into a multivariable logistic regression model. Data completeness was monitored throughout the study. All key variables used in the analysis had minimal or no missing values. No imputation methods were required, and all enrolled participants were included in the final analysis. The sample size calculation was based on an expected prevalence of 0.85 [25] and a margin of error of 0.05. Given that our hospital is expected to perform kidney biopsies on 350 patients during the study period, the required sample size was determined to be 145 [26]. All statistical analyses were conducted using the statistical software package R version 4.4.1 [27], with a significance level (α) set at 5%.

Trial registration: Thai Clinical Trials Registry TCTR20240526005. Registered on 26 May 2024. Retrospectively registered.

## 3. Results

**Figure 1:**
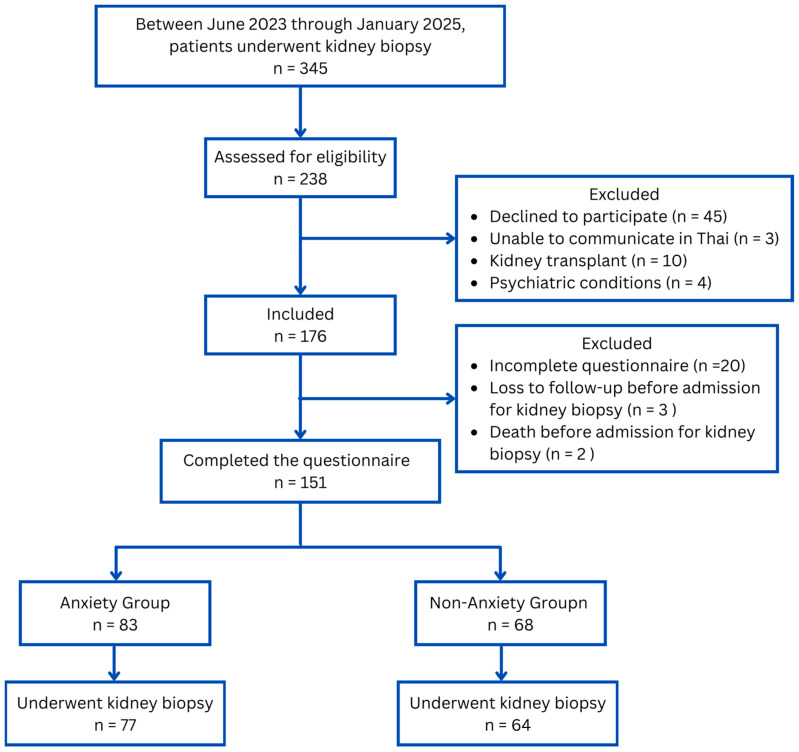
Patient Flow Diagram. Flowchart depicting the selection process of study patients.

Figure 1 illustrates the flow of 345 patients who underwent kidney biopsy between June 2023 and January 2025. Of these, 151 completed the questionnaires, and 141 had their biopsies performed; 10 patients had their biopsies canceled (7 due to severe hypertension and 3 due to dyspnea). Using an STAI-Y1 score ≥ 40 to define anxiety, 55% (83/151) of patients reported experiencing anxiety. Among those with anxiety, 56.6% (47/83) exhibited a high level of anxiety (STAI-Y1 40–49), while 43.4% (36/83) displayed very high anxiety (STAI-Y1 ≥ 50). The mean score on the STAI was 32.0 ± 5.6 for the Non-Anxiety group compared to 47.3 ± 5.8 for the Anxiety group. This difference was statistically significant (*p* < 0.001).

Baseline characteristics (Table 1) were comparable overall between the Anxiety (*n* = 83) and Non-Anxiety (*n* = 68) groups. However, a significantly lower proportion of patients in the anxiety group were married (65.1% vs. 80.9%; *p* = 0.048), and a greater percentage had undergone a previous kidney biopsy (26.5% vs. 8.8%; *p* = 0.010). While baseline SBP, DBP, and pulse rate were similar between the groups, the Anxiety group showed significantly higher blood urea nitrogen levels (30 vs. 21 mg/dL; *p* = 0.001) and serum creatinine levels (1.85 vs. 1.15 mg/dL; *p* = 0.031). There were no significant differences in estimated glomerular filtration rate or hemoglobin levels between the two groups.

### 3.1. Impact of Anxiety on Hemodynamic Parameters

**Figure 2:**
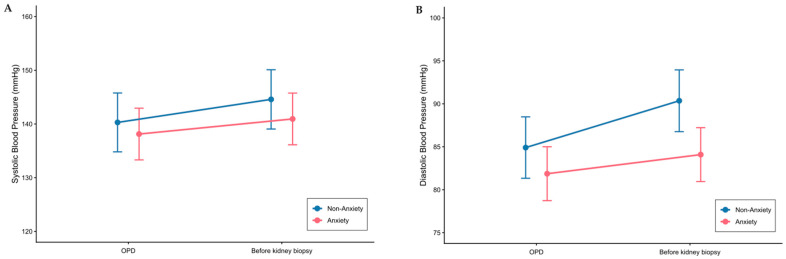
Systolic and Diastolic Blood Pressure Changes in Patients Undergoing Kidney Biopsy, Stratified by Anxiety Status. Least-squares mean changes in systolic blood pressure (SBP; Panel (**A**)) and diastolic blood pressure (DBP; Panel (**B**)) from baseline (outpatient visit) to pre-biopsy (30 min before the procedure), stratified by anxiety status (Anxiety, STAI-Y1 ≥ 40, vs. Non-Anxiety, STAI-Y1 < 40). Error bars represent 95% confidence intervals derived from mixed-effects models adjusted for age, sex, BMI, serum creatinine, and number of antihypertensive drugs.

Table 2 presents the mixed-effects model results evaluating the impact of anxiety on hemodynamic parameters. For SBP, the model estimated an increase of 4.29 mmHg from baseline to pre-biopsy (95% CI −1.66 to 10.22, *p* = 0.159), which was not statistically significant (shown in Figure 2A). Additionally, neither the main effect of anxiety nor the interaction between anxiety and time was statistically significant.

In contrast, DBP significantly increased over time, rising 5.45 mmHg (95% CI 1.47 to 9.43, *p* = 0.008) from baseline to pre-biopsy (shown in Figure 2B). However, neither the main effect of anxiety nor the time-by-anxiety interaction reached statistical significance for DBP. Similarly, no significant effects were observed for pulse rate for time, anxiety, or their interaction.

### 3.2. Factors Associated with Anxiety

Table 3 summarizes the factors related to anxiety among the study participants. Univariate analysis indicated that demographic factors such as female sex, age, and BMI were not significantly associated with anxiety. Similarly, clinical factors, including hypertension, diabetes mellitus, and dyslipidemia, demonstrated no significant associations. In contrast, marital status was significantly associated with anxiety, with married patients showing lower odds (OR 0.44, 95% CI 0.20–0.92, *p* = 0.033) compared to single patients. Furthermore, a history of previous kidney biopsy was significantly linked to higher odds of anxiety (OR 3.73, 95% CI 1.49–10.69, *p* = 0.008), and increased serum creatinine levels were also significantly correlated with anxiety (OR 1.24, 95% CI 1.06–1.50, *p* = 0.014).

After adjusting for potential confounders, multivariate analysis confirmed that serum creatinine remained a significant predictor of anxiety (OR 1.29, 95% CI 1.07–1.59, *p* = 0.012). A history of previous kidney biopsy was independently associated with anxiety (OR 4.28, 95% CI 1.64–12.72, *p* = 0.004). However, marital status showed no association with anxiety.

### 3.3. Post-Biopsy Complications

Among the 141 patients who underwent kidney biopsy, perirenal hematoma was the most common complication, being observed in 43 patients (30.5%); of these, 40 (28.4%) were asymptomatic and 3 (2.1%) were symptomatic. Other complications included angioembolization in 8 patients (5.7%), blood transfusion in 6 (4.3%), prolonged hospitalization in 3 (2.1%), and one death (0.7%). Complication rates did not differ significantly between the Anxiety and Non-Anxiety groups (Table 4).

## 4. Discussion

Our prospective cohort study demonstrates a high prevalence of preprocedural anxiety among patients undergoing kidney biopsy, with 55% of respondents meeting the threshold for clinically significant anxiety. Notably, anxiety did not significantly affect overall hemodynamic parameters; however, DBP increased significantly from the outpatient visit to immediately before the kidney biopsy. Additionally, multivariate analysis identified higher serum creatinine levels and previous kidney biopsy as significant factors associated with anxiety.

To our knowledge, no prospective cohort study has evaluated the prevalence of anxiety in patients undergoing kidney biopsy. A previous randomized trial conducted in Italy examined the effect of music therapy on anxiety in this population, including a total of 80 patients [25]. Notably, all participants in that study had STAI-Y1 scores exceeding 40. This discrepancy likely reflects fundamental differences in study design and population. The previous study was an interventional trial that may have attracted more symptomatic or motivated patients, whereas our observational cohort consecutively enrolled all eligible patients, representing a broader, more heterogeneous population. Additional factors that may have contributed to this difference include the timing of the anxiety evaluation [28,29], socioeconomic status [30], the patient’s country of origin [3,31,32], and clinical practices (e.g., the information provided about the procedure and its associated risks) [33]. Of note, our sample size calculation was based on an expected prevalence of 85% derived from the previous study [25]. The lower-than-expected observed prevalence may have affected the precision of our prevalence estimate.

The physiological response to anxiety involves heightened sympathetic nervous system activity coupled with reduced parasympathetic nervous system activity [25]. Given this physiological basis and previous studies demonstrating that preoperative anxiety is associated with elevated blood pressure in patients awaiting surgery [34], we hypothesized that anxiety would play a significant role in elevating blood pressure before a kidney biopsy. However, our study found no significant impact of anxiety on physiological parameters before the procedure. This finding aligns with previous studies in surgical patients [35,36], though it contrasts with others reporting positive associations [37,38,39].

Several factors may explain these discrepant results. First, our assessment of anxiety 24 h before the biopsy may not have captured the acute anxiety experienced immediately before or during the procedure, as anxiety levels are typically highest when measured closer to the time of intervention [28,29]. Second, our two-point hemodynamic measurement protocol may have missed transient anxiety-related changes; continuous monitoring of blood pressure variability and heart rate variability might provide more sensitive markers of autonomic nervous system activity associated with anxiety [39,40]. Third, concurrent antihypertensive medication use may have masked anxiety’s effects on hemodynamic parameters. Fourth, our sample size was designed primarily to estimate anxiety prevalence rather than to detect differences in hemodynamic parameters across anxiety groups; therefore, the study may have been underpowered to detect clinically meaningful hemodynamic changes.

Anxiety-induced blood pressure elevations, commonly termed white-coat hypertension, can manifest as elevations in both systolic and diastolic blood pressure, with variable patterns depending on the population and measurement conditions [41]. In our study, while both SBP and DBP increased from the outpatient visit to pre-biopsy measurement, only DBP reached statistical significance. Anxiety may preferentially influence diastolic blood pressure in certain contexts, and isolated diastolic hypertension has been reported as a white-coat phenomenon in 29–40% of cases [42]. However, our two-point measurement approach may have been insufficient to capture the full temporal dynamics of anxiety-related hemodynamic changes, particularly transient fluctuations in SBP. As noted above, continuous assessment of blood pressure variability or heart rate variability may represent more appropriate methodologies for evaluating anxiety’s hemodynamic effects [39,40].

The finding that prior kidney biopsy increases anxiety contrasts with surgical literature showing familiarity reduces preoperative anxiety [43]. This paradox may be explained by two main factors. First, unlike surgical procedures in which recovery is often definitive, the need for repeat kidney biopsy may signal disease progression or ongoing kidney pathology, thereby heightening illness-related anxiety and uncertainty. Second, patients may vividly recall negative aspects of previous biopsy experiences, such as pain, discomfort, or prolonged post-procedural bed rest, which may amplify anticipatory anxiety [44]. Future qualitative studies examining patient perspectives on repeat kidney biopsy could provide valuable insights into this phenomenon. 

We found no significant association between anxiety status and post-biopsy complications; however, our study was underpowered to detect such differences. The clinical impact of preprocedural anxiety on biopsy-related adverse events, therefore, remains uncertain and warrants further investigation in larger cohorts.

Our findings have several clinical implications. First, given the 55% prevalence of preprocedural anxiety, which is comparable to rates of 48% reported in patients awaiting surgical procedures [45], routine screening using brief validated instruments should be considered for all kidney biopsy patients. Second, patients with elevated serum creatinine or a history of previous biopsies should be identified as high-risk for anxiety and offered targeted interventions such as enhanced patient education, relaxation techniques, or the consideration of anxiolytic premedication. Finally, developing standardized anxiety management protocols specific to kidney biopsy could improve patient-centered care in nephrology.

Our study has several limitations. First, we did not use objective measures of anxiety, such as electrical skin potential [46], urine catecholamines [47], or plasma catecholamines [48]. Instead, we relied solely on the STAI-Y1, which is inherently subjective. However, the STAI-Y1 is the most widely used instrument for measuring the severity of anxiety [24]. Second, as with all observational studies, causal inference cannot be definitively established. However, we adjusted for a comprehensive set of relevant demographic and clinical variables, including sex, age, comorbidities, educational level, and laboratory measurements. Residual confounding, such as prior negative procedural experiences, health literacy, and psychological factors, including fear and depression, cannot be excluded. Additionally, psychiatric conditions were identified based on medical records and patient self-report rather than formal psychiatric evaluation, which may have resulted in underdetection of undiagnosed conditions and potential selection bias. Third, blood pressure and pulse rate were measured at only two time points, which may not have been sufficient to capture transient sympathetic activation or white-coat effects. Finally, our study was conducted in a single tertiary center in Thailand, a middle-income country, which may limit its generalizability to other settings. Center-specific factors could also have influenced anxiety levels, including institutional experience and communication practices during the preparation phase. At our center, preprocedural education was provided by a trained nurse coordinator; however, approaches may vary across institutions, with some centers involving the proceduralist or other physicians in patient preparation. These variations in provider communication may differentially affect patient anxiety levels and could account for differences in anxiety prevalence across centers. Despite these limitations, a key strength of our study is that it is the first prospective cohort study specifically designed to assess the prevalence of anxiety before a kidney biopsy.

## 5. Conclusions

In conclusion, this study highlights the high prevalence of preprocedural anxiety among patients undergoing kidney biopsy, with higher serum creatinine levels and previous biopsy history identified as key contributing factors. While anxiety did not significantly impact overall hemodynamic parameters, the observed increase in diastolic blood pressure from baseline to pre-biopsy suggests a potential physiological stress response. These findings underscore the need for structured preprocedural anxiety assessments. Future research with larger sample sizes and continuous hemodynamic monitoring should explore whether preprocedural anxiety influences hemodynamic change and post-biopsy bleeding complications.

## Figures and Tables

**Table 1:** Baseline Demographic and Clinical Characteristics by Anxiety Status.

Variable	Anxiety (STAI-Y1 ≥ 40; n = 83)	Non-Anxiety (STAI-Y1 < 40; n = 68)	*p*
**Demographics**			
Age, years	42 [30.5–54]	45 [33.2–54]	0.304
Female sex, n (%)	45 (54.2)	41 (60.3)	0.342
BMI, kg/m^2^	23.3 [21.0–27.7]	25.1 [22.0–28.0]	0.552
Married, n (%)	54 (65.1)	55 (80.9)	0.048
Education, n (%)			0.975
Illiterate	2 (2.4)	2 (2.9)	
Elementary school	25 (30.1)	19 (27.9)	
High school	37 (44.6)	34 (50.0)	
Bachelor’s degree	17 (20.5)	12 (17.6)	
Master’s degree	2 (2.4)	1 (1.5)	
Unemployed, n (%)	33 (39.8)	27 (39.7)	1.000
**Clinical history**			
Previous kidney biopsy, n (%)	22 (26.5)	6 (8.8)	0.010
Indication for biopsy, n (%)			0.525
Acute nephritis	22 (27.2)	15 (22.1)	
Nephrotic syndrome	48 (59.3)	42 (61.8)	
Non-nephrotic proteinuria	8 (9.9)	5 (7.4)	
Other	3 (3.7)	6 (8.8)	
**Comorbidities, n (%) ^a^**			
Hypertension	38 (46.9)	36 (53.7)	0.509
Diabetes mellitus	25 (30.9)	18 (26.9)	0.725
Dyslipidemia	17 (21.0)	19 (28.4)	0.520
Chronic kidney disease	35 (43.2)	22 (32.8)	0.262
Systemic lupus erythematosus	18 (22.2)	19 (28.4)	0.505
**Medications**			
Number of antihypertensive drugs ^a^	2 [1–3]	2 [1–4]	0.714
Beta blocker use, n (%) ^a^	18 (22.2)	14 (20.9)	1.000
**Hemodynamic parameters**			
Systolic BP, mmHg	135 [126–153]	140 [128–151]	0.383
Diastolic BP, mmHg	81 [71–89]	83 [72–93]	0.587
Pulse rate, beats/min	80 [70–93]	80 [71–92]	0.940
**Laboratory values**			
Blood urea nitrogen, mg/dL	30 [19–51]	21 [15–30]	0.001
Serum creatinine, mg/dL	1.85 [0.88–4.25]	1.15 [0.85–2.43]	0.031
eGFR, mL/min/1.73 m^2^	43.7 [12.9–91.3]	55.2 [25.8–85.7]	0.231
Hemoglobin, g/dL	10.2 [8.7–12.0]	10.4 [8.9–13.0]	0.256

Data are presented as median [interquartile range] or number (percent). Abbreviations: BMI, body mass index; BP, blood pressure; eGFR, estimated glomerular filtration rate; STAI, State-Trait Anxiety Inventory. ^a^ Denominators for comorbidity and medication variables are n = 81 (Anxiety group) and n = 67 (Non-Anxiety group) due to missing data.

**Table 2:** Mixed-Effects Model Results for Hemodynamic Parameters.

Variable	SBP, mmHg (95% CI) ^†^	*p* Value	DBP, mmHg (95% CI) ^†^	*p* Value	Pulse Rate, bpm (95% CI) ^‡^	*p* Value
**Time**	4.29 (−1.66 to 10.22)	0.159	5.45 (1.47 to 9.43)	0.008	−0.42 (−5.21 to 4.37)	0.863
**Anxiety**	−2.16 (−9.35 to 5.02)	0.562	−3.04 (−7.72 to 1.65)	0.212	−1.55 (−7.02 to 3.91)	0.584
**Time × Anxiety**	−1.48 (−9.37 to 6.44)	0.715	−3.23 (−8.52 to 2.07)	0.235	0.17 (−6.21 to 6.54)	0.958

Note: Values are presented as the estimated effect (with 95% confidence intervals) and corresponding *p* values. Abbreviations: SBP, systolic blood pressure; DBP, diastolic blood pressure; bpm, beats per minute. ^†^ SBP and DBP models were adjusted for age, sex, BMI, serum creatinine, and number of antihypertensive drugs. ^‡^ Pulse rate models were adjusted for age, sex, BMI, serum creatinine, and beta-blocker use.

**Table 3:** Factors associated with anxiety among the study patients: Univariate and Multivariate Analysis.

Variable	Univariate Analysis			Multivariate Analysis		
	OR	95% CI	*p*	OR	95% CI	*p*
**Demographics**						
Female sex	0.78	0.41–1.49	0.453	0.91	0.43–1.92	0.812
Age, per year	0.99	0.97–1.01	0.299	0.99	0.97–1.02	0.499
BMI, per kg/m^2^	0.99	0.94–1.05	0.799	—	—	—
**Marital status**						
Single	1.00	(ref)				
Married	0.44	0.20–0.92	0.033	0.59	0.23–1.44	0.252
**Education**						
Illiterate	1.00	(ref)				
Elementary school	1.32	0.15–11.79	0.793	—	—	—
High school	1.09	0.12–9.48	0.934	—	—	—
Bachelor’s degree	1.42	0.15–13.20	0.744	—	—	—
Master’s degree	2.00	0.09–69.06	0.661	—	—	—
**Employment status**						
Employed	1.00	(ref)				
Unemployed	1.00	0.52–1.94	0.995	—	—	—
**Comorbidities**						
Hypertension	0.76	0.40–1.45	0.409	—	—	—
Diabetes mellitus	1.22	0.60–2.51	0.594	—	—	—
Dyslipidemia	0.72	0.34–1.55	0.403	—	—	—
**Clinical factors**						
Previous kidney biopsy	3.73	1.49–10.69	0.008	4.28	1.64–12.72	0.004
**Indication for biopsy**						
Other	1.00	(ref)				
Acute nephritis	2.93	0.67–15.67	0.169	—	—	—
Nephrotic syndrome	2.29	0.57–11.34	0.263	—	—	—
Non-nephrotic proteinuria	3.20	0.57–21.47	0.200	—	—	—
**Laboratory values**						
Serum creatinine, per mg/dL	1.24	1.06–1.50	0.014	1.29	1.07–1.59	0.012
UPCR, per g/g	1.00	0.99–1.00	0.930	—	—	—

Abbreviations: BMI, body mass index; CI, confidence interval; OR, odds ratio; UPCR, urine protein-creatinine ratio. — indicates variable not included in multivariate model.

**Table 4:** Post-Biopsy Complications by Anxiety Status.

Complication	Non-Anxiety (STAI-Y1 < 40), n = 64	Anxiety (STAI-Y1 ≥ 40), n = 77	Overall n = 141	*p* Value
**Perirenal hematoma**				0.549
None	42 (65.6%)	56 (72.7%)	98 (69.5%)	
Asymptomatic	20 (31.2%)	20 (26.0%)	40 (28.4%)	
Symptomatic	2 (3.1%)	1 (1.3%)	3 (2.1%)	
**Angioembolization**	4 (6.3%)	4 (5.2%)	8 (5.7%)	1.000
**Prolonged admission**	0 (0%)	3 (3.9%)	3 (2.1%)	0.251
**Blood transfusion**	3 (4.7%)	3 (3.9%)	6 (4.3%)	1.000
**Death**	0 (0%)	1 (1.3%)	1 (0.7%)	1.000

Data are presented as numbers (percent). Abbreviations: STAI-Y1, State-Trait Anxiety Inventory, Form Y1.

## Data Availability

The datasets analyzed during the current study are available from the corresponding author upon reasonable request.

## Abbreviations

The following abbreviations are used in this manuscript:

AVF	Arteriovenous Fistula
BMI	Body Mass Index
BP	Blood Pressure
CI	Confidence Interval
CKD	Chronic Kidney Disease
DBP	Diastolic Blood Pressure
eGFR	Estimated Glomerular Filtration Rate
MMRM	Mixed-Effects Model for Repeated Measures
OR	Odds Ratio
SBP	Systolic Blood Pressure
STAI-Y1	State–Trait Anxiety Inventory, Form Y1
